# Disturbance and Recovery of Salt Marsh Arthropod Communities following BP Deepwater Horizon Oil Spill

**DOI:** 10.1371/journal.pone.0032735

**Published:** 2012-03-07

**Authors:** Brittany D. McCall, Steven C. Pennings

**Affiliations:** Department of Biology and Biochemistry, University of Houston, Houston, Texas, United States of America; Biodiversity Insitute of Ontario - University of Guelph, Canada

## Abstract

Oil spills represent a major environmental threat to coastal wetlands, which provide a variety of critical ecosystem services to humanity. The U.S. Gulf of Mexico is a hub of oil and gas exploration activities that historically have impacted intertidal habitats such as salt marsh. Following the BP Deepwater Horizon oil spill, we sampled the terrestrial arthropod community and marine invertebrates found in stands of *Spartina alterniflora*, the most abundant plant in coastal salt marshes. Sampling occurred in 2010 as oil was washing ashore and a year later in 2011. In 2010, intertidal crabs and terrestrial arthropods (insects and spiders) were suppressed by oil exposure even in seemingly unaffected stands of plants; however, *Littoraria* snails were unaffected. One year later, crab and arthropods had largely recovered. Our work is the first attempt that we know of assessing vulnerability of the salt marsh arthropod community to oil exposure, and it suggests that arthropods are both quite vulnerable to oil exposure and quite resilient, able to recover from exposure within a year if host plants remain healthy.

## Introduction

Oil spills represent a major environmental threat to coastal wetlands, which provide a variety of critical ecosystem services to humanity. Benefits of coastal wetlands include protection from shoreline erosion, regulation of gasses and nutrients, provision of biological diversity, support of primary and secondary production, provision of nutrient and organismal subsidies to adjacent ecosystems, and support of fishery and ecotourism industries [1], [2], [3]. Rising sea level, coastal subsidence, hurricanes, invasive species, erosion, coastal land use change, and oil spills threaten the health of this ecosystem [4]. Because coastal marshes are low-energy and anoxic environments, dispersal and decomposition of oil is slow, and oil incorporated into the sediment can persist for many years [5], [6], [7]. This persistent oil may affect flora and fauna for generations and impede recovery of the system. There are over 3,000 active oil & gas production platforms in U.S. Outer Continental Shelf of the Gulf of Mexico, where coastal marshes are the dominant intertidal habitat [8], [9]. Thus, it is important to accurately identify consequences of oil spills in these habitats.

Known effects of oil contamination in salt marshes include alteration of soil properties and negative impacts on plant populations, benthic invertebrate populations, and fish and invertebrate populations that use salt marshes as feeding or nursery grounds [5], [10], [11], [12]. There is much, however, that we do not know about how oil affects coastal wetlands. Many of the relevant studies have for obvious reasons been done in the laboratory, with a limited range of taxa, a short duration, and a failure to mimic field conditions such as tides and waves that might affect the fate and impact of oil [13], [14], [15]. Field studies have had longer durations and avoid possible laboratory artifacts, but both field and lab studies have focused on abiotic conditions, plants, and marine invertebrates [16], [17], [18]. The salt marsh “terrestrial arthropod” community, which includes a diversity of insects and spiders and which represents an important trophic link to both terrestrial and marine vertebrates (such as birds and juvenile fishes [19]), has been neglected.

Following the BP Deepwater Horizon oil spill, we sampled the terrestrial arthropod community found in stands of *Spartina alterniflora*, the most abundant plant in coastal salt marshes. We also sampled marine invertebrates for comparison. Sampling occurred in 2010 as oil was washing ashore and again a year later in 2011. The terrestrial arthropods found in coastal marshes dominated by *Spartina alterniflora* encompass about 100 species that include major herbivores, predators, parasitoids, and detritivores [20]. This project is the first attempt that we know of to assess impacts of oil spills on this arthropod community. Our results indicate that the terrestrial arthropods of coastal salt marshes are both quite vulnerable to oil exposure and quite resilient, able to recover from exposure within a year if host plants remain healthy.

## Materials and Methods

**Figure 1:**
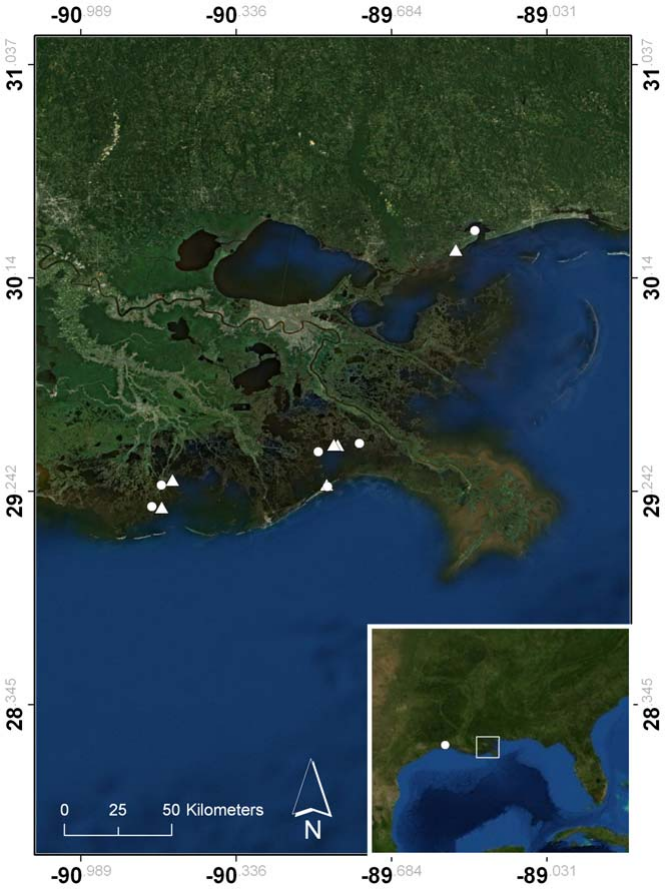
Location of sampling sites. Circles indicate control sites; triangles indicate oiled sites. Sampling dates and GPS coordinates may be found in Table S1.

**Study Sites and System.** Field work was conducted in Louisiana and Mississippi at 10 sites in 2010 and 12 sites in 2011 (Figure 1, Table S1). Sites were chosen in 2010 based on where oil had made landfall. In 2011, the most remote control site (C1) was replaced with one closer to the oiled sites (C7), and two additional oiled sites (O5, O6) and an associated control (C6) were added to increase replication. Most field sites required no permits as collections were done on public land. Permits were obtained from the US Fish and Wildlife Service for Sabine NWR and Bon Secour NWR; the Louisiana Office of State Parks for Grand Isle State Park and Grand Isle Oiled; and the Coastal Preserves Program of the Mississippi Department of Marine Resources for Bay St. Louis and Bay St. Louis Oiled. All sites were exposed to regular flooding by seawater and were characterized by salt marsh vegetation. The vegetation at each site consisted of a large monospecific stand of *Spartina alterniflora* at lower marsh elevations that transitioned into high marsh vegetation dominated by pure stands or mixtures of *Juncus roemerianus*, *Spartina patens*, *Distichlis spicata*, *Sarcocornia* spp., *Batis maritima*, *Borrichia frutescens*, *Avicennia germinans*, or *Scirpus* spp., depending on the site.

We focused on the terrestrial arthropod community found in stands of *Spartina alterniflora* (henceforth *Spartina*) because it is the most abundant plant in salt marshes along the Gulf Coast of the United States and its arthropod community has been well studied [21], [22], [23], [24], [25], [26]. Also, because *Spartina* occupies the lowest elevations in coastal marshes, this plant and its associated fauna were most affected by the oil spill (authors personal observations). Arthropod samples collected in this study were sorted in the laboratory to 22 focal taxa (Table S2) that represented the “resident” community that lives within the *Spartina* marsh. Taxa such as dragonflies, mosquitoes, and thrips (∼30 percent of total) were excluded because they either represented transient species or were extremely rare in the community. To reduce the dimensionality of the data set and because some taxa were variable in density across sites, we grouped the most common resident taxa into five feeding guilds: sucking herbivores, stem-boring herbivores, predators, parasitoids and detritivores. For comparison, we also sampled intertidal crabs and *Littoraria* snails, common marine invertebrate taxa.

**Figure 2:**
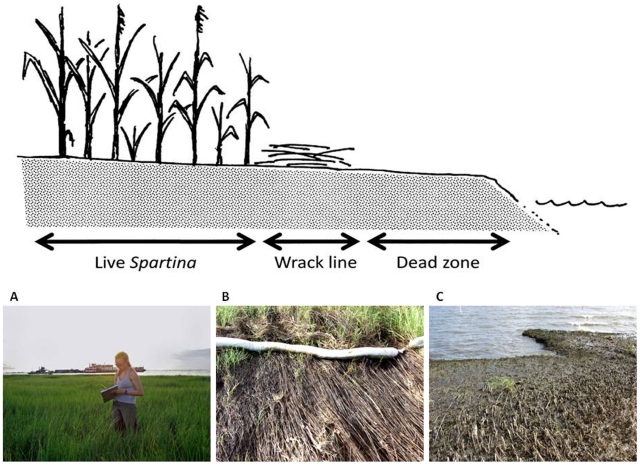
Typical conditions at oiled sites. At the leading edge of the marsh, there was C) a zone up to 5 m wide of bare mud where all vegetation had died. Behind this was B) a zone of wrack up to 2 m wide consisting of heavily-oiled plant debris. Behind this was A) an extensive zone of live and apparently healthy *Spartina alterniflora*. Oil sheens were visible on the soil surface within the live *Spartina* zone. At some oiled sites, the dead zone and wrack line were absent, but oil sheens were visible within the live *Spartina* zone. Sampling at both oiled and control sites was done at least 2 m inside the live *Spartina* zone.

At each site, we characterized abiotic conditions, the plant and arthropod community, and the abundance of two common marine invertebrate taxa using transect sampling. We located a single 100 m transect at each site, 1–2 m into the *Spartina* zone from the lower edge of the marsh, running parallel to the water's edge (Figure 2). The presence of oil was visually confirmed at all of the oiled sites. At many of the oiled sites, the leading edge of the marsh consisted of a band of heavily-oiled, dead *Spartina* up to 5 m wide. If a dead area was present, transects were sampled behind it, 1–2 m into living *Spartina*. The plants that we sampled at oiled sites were often partially coated with oil at the base of the stem, but otherwise appeared healthy. Oil chemistry was not measured. Transects were sampled in August of 2010 and 2011. For each variable, subsamples collected along each transect were pooled, yielding a single value for each site on each date that was used in statistical analyses.

A soil sample (10 cm deep) was collected at four locations along each transect and pooled. The soil sample was weighed wet and then air dried until returned to the laboratory. In the lab, we dried soils at 60°C for 4 days, measured soil water content gravimetrically, and ground and mixed dry soils with a mortar and pestle. Dried soils were rehydrated with a known volume of distilled water, the salinity measured after 24 h, and back-calculated to the original water content to estimate initial pore water salinity. Soil organic content was measured as loss on ignition in a muffle furnace at 440°C for 24 hr.

The vegetation was characterized at four locations along each transect. The percent cover of live *Spartina* plants and of dead plant material, a combination of wrack (dead stems) and thatch (dead leaves), was measured with a 0.5×0.5 m quadrat divided into 100 cells by monofilament line. Live *Spartina* or dead plant material was scored in each cell as present or absent. We recorded the height of the tallest *Spartina* plant in each quadrat, and collected the third leaf from the top of two plants adjacent to each quadrat. The leaves were air-dried until returned to the laboratory, where they were lyophilized for 72 hr, ground with a Spex 8000 M Mixer/Mill, and analyzed for nitrogen content at the UGA Chemical Analysis Lab.

We sampled the *Spartina* arthropod community with a D-Vac suction sampler [27], [28], taking eight, ten-second suction samples (0.0706 m^2^ suction head) spaced evenly along each transect, for a total area of 0.56 m^2^. Arthropod samples were stored in ethanol and sorted in the laboratory. We also characterized marine invertebrate abundance (number m^−2^) along each transect with six quadrats in 2010 and four in 2011. In each quadrat, we counted the number of *Littoraria* snails and the number of crab burrows. By far, the most abundant intertidal crabs at these sites were fiddler crabs, *Uca* spp.

Data were analyzed with ANOVA, with oil (present or absent) and year as the main effects. “Site” was not included as a factor in the analysis because the set of sites sampled differed slightly between years. We used the standard p-value cutoff of 0.05 to indicate statistical significance. However, because the number of replicate sites that we studied was modest and because we were concerned about falsely rejecting the hypothesis that oil exposure affected our response variables, we considered p-values between 0.05 and 0.10 as marginally significant and indicative of strong trends. Raw data have been submitted to the GCE-LTER data catalogue (http://gce-lter.marsci.uga.edu/public/data/data.htm).

## Results

Abiotic conditions and plant variables were similar between oiled and control sites (Table 1). Soil organic content, water content, and soil salinity did not differ between oiled and control sites or between years. Vegetation variables differed slightly among year and oiled treatments (Table 1). *Spartina* leaf nitrogen content was higher in oiled sites and slightly higher in 2010 than 2011; however, *Spartina* plants did not differ in height due to oil impact or year. There was no effect of oil on the percent cover of *Spartina* plants or dead material; however, the percent cover of both increased in both types of sites from 2010 to 2011. There were no significant interactions between year and oil for any plant community variables.

**Table 1:** Measurements of soil and vegetation at oiled and control sites (means ± SE).

Variable	Control 2010	Oiled 2010	Control 2011	Oiled 2011	ANOVA p-values		
	(n = 6)	(n = 4)	(n = 6)	(n = 6)	Oil	Year	Oil*Year
Soil % Organic	15.14±3.79	12.97±5.41	13.29±2.85	15.75±4.43	0.97	0.91	0.58
Soil % Water	59.72±6.23	50.45±9.68	59.59±5.67	56.59±7.03	0.39	0.67	0.66
Soil Salinity	24.78±3.18	14.69±4.57	20.04±6.63	27.26±5.09	0.79	0.47	0.12
Leaf Nitrogen Content	1.26±0.05	1.59±0.23	1.16±0.068	1.33±0.092	0.02	0.098	0.44
Live *Spartina* Height	94.21±4.93	105.69±5.37	89.46±9.69	95.54±6.27	0.24	0.32	0.72
*Spartina* %Cover	93.58±2.07	87.81±7.71	97.17±0.98	96.5±0.53	0.30	0.057	0.41
Dead Material %Cover	60.75±11.82	57.25±14.29	97.33±1.35	89.08±5.28	0.52	0.001	0.79

Data were analyzed with ANOVA; p-values for the main effects of oil and year, and their interaction, are shown.

**Figure 3:**
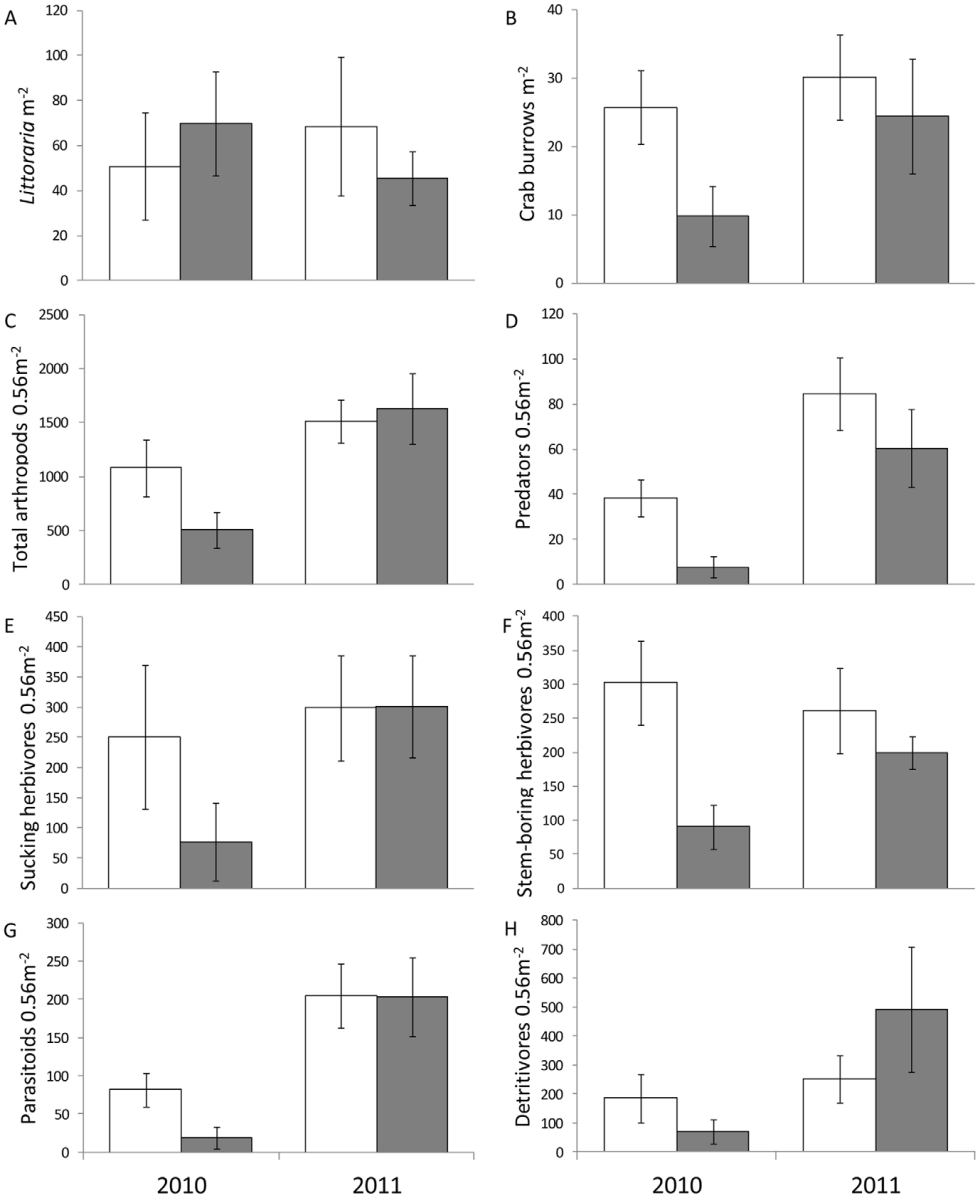
Densities of marine invertebrates and terrestrial arthropods at oiled and control sites in 2010 and 2011. A) *Littoraria* m^−2^; B) crab burrows m^−2^; C) total terrestrial arthropods 0.56 m^−2^ (not including crustaceans); D) predators 0.56 m^−2^; E) sucking herbivores 0.56 m^−2^; F) stem-boring herbivores 0.56 m^−2^; G) parasitoids 0.56 m^−2^; and H) detritivores 0.56 m^−2^. Open bars indicate control sites; filled bars indicate oiled sites. Data are means ±1 SE.

The density of the marine snail, *Littoraria*, was not affected by oil or sampling year (Table 2, Figure 3). The density of crab burrows was higher in control sites and higher (p = 0.07) in 2011 than 2010. Although the interaction between oil and year was not significant, the lowest density of crab burrows was found in 2010 at oiled sites.

**Table 2:** Summary of ANOVA results (d.f., F statistics, and p-values) for invertebrate groups.

Variable	Oil			Year			Oil*Year		
	d.f., error	F	p-value	d.f., error	F	p-value	d.f., error	F	p-value
*Littoraria* m^−2^	1, 18	0.01	0.93	1, 18	0.02	0.89	1, 18	0.76	0.39
Crab Burrows m^−2^*	1, 16	5.86	0.02	1, 16	3.66	0.074	1, 16	2.04	0.17
Total Arthropods 0.56 m^−2^*	1, 18	3.06	0.097	1, 18	12.33	0.002	1, 18	3.15	0.093
Predators 0.56 m^−2^*	1, 18	13.8	0.001	1, 18	23.4	0.0001	1, 18	6.09	0.02
Sucking Herbivores 0.56 m^−2^*	1, 18	6.17	0.02	1, 18	10.3	0.004	1, 18	5.01	0.03
Stem-Boring Herbivores 0.56 m^−2^*	1, 18	8.53	0.009	1, 18	2.66	0.12	1, 18	5.25	0.03
Parasitoids 0.56 m^−2^	1, 18	0.65	0.43	1, 18	14.9	0.001	1, 18	0.60	0.45
Detritivores 0.56 m^−2^	1, 18	0.21	0.65	1, 18	3.15	0.093	1, 18	1.65	0.22

Sample sizes are as in Table 1. Asterisks indicate variables that were natural log transformed before analysis.

The terrestrial arthropod community was suppressed by 50% at oiled sites in 2010, but largely recovered in 2011 (oil*year interaction, p = 0.093, Table 2, Figure 3). Similar patterns were observed for the five feeding guilds examined. Predators, sucking herbivores, stem-boring herbivores, parasitoids, and detritivores all tended to be suppressed at oiled sites by 25% to 50% in 2010 and to have recovered in 2011. Although all five feeding guilds showed the same general pattern, the interaction between oil and year was only statistically significant for three guilds (predators, sucking herbivores and stem-boring herbivores) due to modest site replication and moderate variability among sites.

## Discussion

To the best of our knowledge, this is the first study assessing vulnerability of salt marsh arthropod communities to oil spills. We sampled in stands of *Spartina alterniflora* that appeared healthy and unaffected by oil, though an oil sheen was visible at the soil surface. Moreover, soil and vegetation characteristics were very similar between oiled and control sites. Nevertheless, we found that the invertebrate community living in these stands was strongly affected by oil exposure. The invertebrate community recovered within one year, however, which is consistent with previous studies showing that salt marshes in this region are fairly resilient to oil disturbance [6], [15], [29].

We found no difference in soil properties due to the presence of oil and only slight differences in the vegetation. Oil contamination can alter chemical and physical soil properties, such as porosity and redox potential [12], but for the variables we measured there were no significant differences between sites. There were also no differences between oiled and control sites in plant height, live plant cover, or dead material cover. This is likely because we sampled several meters into the marsh from the area of heaviest oil exposure. The elevated leaf nitrogen content that we observed at oiled sites is perplexing. Oil contamination may reduce plant photosynthetic rate, transpiration rate, carbon dioxide fixation, and gas-exchange [11]; however, there is no information that we are aware of indicating oil exposure should increase leaf nitrogen content. This relationship is intriguing and deserves more attention. Overall, sites with and without oil impact had similar abiotic conditions and vegetation characteristics, suggesting that differences in the invertebrate community we documented were due to oil exposure, not to underlying variation in abiotic conditions or vegetation structure.

Although snail densities did not vary between oiled and control sites, crab burrows were suppressed at oiled sites. Previous studies have shown that oil exposure negatively affects intertidal crabs. In New England, exposure to oil led to shallower burrows and, over time, to lower population densities of *Uca* fiddler crabs [30]. An oil spill in an intertidal mangrove community in Australia similarly led to lower crab burrow densities; however, crabs at this site recovered within six months [31]. Crab burrows may be beneficial for wetland recovery because they increase oxygenation of soils, thereby promoting oil degradation [32].

We observed that exposure to crude oil strongly suppressed the *Spartina* terrestrial arthropod community, but that the community had largely recovered after a year. The mechanisms by which oil harmed arthropods were not assessed, but the arthropods were likely exposed to both volatile hydrocarbons and direct contact with oil. We know of no information regarding toxicity of volatile hydrocarbons, which are considered the most toxic component of crude oil, to arthropods; however, direct exposure to oil may affect feeding and oviposition behaviors, alter cuticle permeability, disrupt gas exchange, cause desiccation, and destroy cell membrane structure and function [33], [34]. Regardless of the mechanism, it is clear that oil exposure negatively impacts this community.

In 2011, one year after initial oil exposure, all arthropod groups had rebounded to densities similar to those at control sites. This rapid recovery may be due to the unique characteristics of the BP Deepwater Horizon spill that allowed for considerable weathering and degradation of oil before impact [35]. Alternatively, arthropods might quickly recover regardless of spill characteristics through migration from adjacent unaffected stands of plants as the most toxic, volatile fractions of oil degrade or evaporate and as residual oil becomes incorporated into the sediments. While sediment-dwelling organisms may be exposed to buried oil for decades [5], [36], arthropods that live in plants are unlikely to come into contact with oil once it is buried in sediments, and so might only be affected indirectly through effects of oil on plant vigor and chemistry. Because natural disturbances in coastal wetlands are common [37], [38], [39], arthropods must routinely re-colonize disturbed patches, and this has likely produced a faunal community that is resilient to many kinds of periodic disturbance. Thus, a quick recovery may be the norm for healthy salt marsh habitats if the disturbance event is under some critical threshold and if there are adjacent unaffected habitats that can serve as a source for colonists.

The BP Deepwater Horizon spill was distinct from most other major oil spills in that the well was far offshore, allowing a long period of time for the oil to weather before it reached the shoreline; the spill occurred in a subtropical region where there is an almost year-round growing season and a high potential for year-round microbial activity that could degrade the oil; and there was an unprecedented 1.84 million gallons of dispersant applied to enhance natural degradation [35]. Because these unique characteristics likely influenced the effects of the spilled oil on the salt marsh community, we guard against uncritically extrapolating our results to other spills with different traits and geographic context. Our data, however, suggest two important conclusions. Firstly, we cannot assume that there is no impact to a salt marsh based on observations that plants seem unaffected, because fauna may be more sensitive than plants and may be suppressed even in areas where plants show no visual signs of harm. Secondly, salt marsh terrestrial arthropods are able to quickly recover from episodic disturbances. While we did not experimentally address the conditions required for this kind of rapid recovery, it is likely that healthy host plants and a nearby source of colonists are necessary. To accurately identify the consequences of oil exposure on coastal systems, multiple types of fauna along with the more obvious plants and large vertebrates must be considered.

## Supporting Information

Table S1 **Study sites and sampling dates.**
(DOC)

Table S2 **Taxonomic categories used in sorting DVAC arthropod collections.**
(DOC)
