# Supplementary material for: Disturbance and Recovery of Salt Marsh Arthropod Communities following BP Deepwater Horizon Oil Spill
Source: PLoS One. 2012 Mar 7;7(3):e32735. doi: 10.1371/journal.pone.0032735 (PMC3296729; doi:10.1371/journal.pone.0032735)
Supplement: Table S1 — Study sites and sampling dates. (DOC) [file pone.0032735.s001.doc]

Disturbance and recovery of salt marsh arthropod communities following BP Deepwater Horizon oil spill: Supporting Information

Brittany D. McCall and Steven C. Pennings

Table S1. Study sites and sampling dates.

| Site Name | Code | Latitude | Longitude | Sampling Dates | | |
| --- | --- | --- | --- | --- | --- | --- |
|  |  | GPS (DD, M.MM) | GPS (DD, M.MM) | day | month | year |
| Sabine NWR Control | C 1 | N29 53.340 | W93 24.056 | 17 | 8 | 2010 |
| Cocodrie LA Control | C 2 | N29 16.123 | W90 39.047 | 18 | 8 | 2010 |
| Cocodrie LA Control | C 2 | N 29 16.139 | W 90 39.023 | 17 | 8 | 2011 |
| Terrebonne Bay Control | C 6 | N 29 10.733 | W 90 41.397 | 18 | 8 | 2011 |
| Terrebonne Bay Oil 1 | O 5 | N 29 17.418 | W 90 36.216 | 18 | 8 | 2011 |
| Terrebonne Bay Oil 2 | O 6 | N 29 10.481 | W 90 38.952 | 18 | 8 | 2011 |
| Grand Isle State Park Control | C 3 | N29 15.727 | W89 57.158 | 20 | 8 | 2010 |
| Grand Isle State Park Control | C 3 | N 29 15.728 | W 89 57.159 | 20 | 8 | 2011 |
| Grand Isle Oil | O 1 | N29 16.163 | W89 57.329 | 20 | 8 | 2010 |
| Grand Isle Oil | O 1 | N 29 16.164 | W 89 57.328 | 20 | 8 | 2011 |
| Barataria Islands Oil 1 | O 2 | N29 26.726 | W89 55.773 | 19 | 8 | 2010 |
| Barataria Islands Oil 1 | O 2 | N 29 26.738 | W 89 55.721 | 19 | 8 | 2011 |
| Barataria Islands Oil 2 | O 3 | N29 26.198 | W89 54.598 | 19 | 8 | 2010 |
| Barataria Islands Oil 2 | O 3 | N 29 26.197 | W 89 54.595 | 19 | 8 | 2011 |
| Barataria Islands Control 1 | C 4 | N29 24.569 | W89 59.494 | 19 | 8 | 2010 |
| Barataria Islands Control 1 | C 4 | N 29 24.563 | W 89 59.493 | 19 | 8 | 2011 |
| Barataria Islands Control 2 | C 7 | N 29 26.624 | W 89 49.05 | 19 | 8 | 2011 |
| Bay St. Louis MS Control | C 5 | N30 20.298 | W89 20.014 | 23 | 8 | 2010 |
| Bay St. Louis MS Control | C 5 | N 30 20.305 | W 89 19.999 | 16 | 8 | 2011 |
| Bay St. Louis MS Oil | O 4 | N30 15.437 | W89 24.867 | 23 | 8 | 2010 |
| Bay St. Louis MS Oil | O 4 | N 30 15.466 | W 89 24.769 | 16 | 8 | 2011 |
