# Supplementary material for: Disturbance and Recovery of Salt Marsh Arthropod Communities following BP Deepwater Horizon Oil Spill
Source: PLoS One. 2012 Mar 7;7(3):e32735. doi: 10.1371/journal.pone.0032735 (PMC3296729; doi:10.1371/journal.pone.0032735)
Supplement: Table S2 — Taxonomic categories used in sorting DVAC arthropod collections. (DOC) [file pone.0032735.s002.doc]

Disturbance and recovery of salt marsh arthropod communities following BP Deepwater Horizon oil spill: Supporting Information

Brittany D. McCall and Steven C. Pennings

Table S2. Taxonomic categories used in sorting DVAC arthropod collections.

| Feeding Guild | Taxa | Order | Family |
| --- | --- | --- | --- |
| Herbivores (sap-sucking) |  |  |  |
|  | *Trigonotylus* | Hemipteran | Miridae |
|  | Leafhoppers | Hemipteran | Cicadellidae |
|  | *Prokelisia* planthoppers (2 spp) | Hemipteran | Delphacidae |
|  | *Delphacodes* planthoppers | Hemipteran | Delphacidae |
|  | *Megamealus* planthoppers | Hemipteran | Delphacidae |
|  | Immature planthoppers | Hemipteran | Delphacidae |
|  | Other Planthoppers | Hemipteran | Misc. |
|  | *Ischnodemus* | Hemipteran | Blissidae |
| Herbivores (stem-boring) |  |  |  |
|  | *Chaetopsis* Fly | Diptera | Ulidiidae |
|  | Chloropidae | Diptera | Chloropidae |
|  | Cecidomyiidae | Diptera | Cecidomyiidae |
|  | Dolichopodidiae | Diptera | Dolichopodidae |
| Predators |  |  |  |
|  | *Tytthus vagus* | Hemiptera | Miridae |
|  | *Naemia* sp*.* | Coleoptera | Coccinellidae |
|  | Lycosid spiders | Araneae | Lycosidae |
|  | Salticid spiders | Araneae | Salticidae |
|  | Clubionid spiders | Araneae | Clubionidae |
|  | Tetragnatha spiders | Araneae | Tetragnathidae |
|  | Linyphiid spiders | Araneae | Linyphiidae |
|  | Other Spiders | Araneae | Misc. |
| Parasitoids |  |  |  |
|  | Wasps | Hymenoptera | Misc. |
| Detritivores |  |  |  |
|  | Collembola | Collembola | Misc. |
